# Designing an Optimum Fiscal Policy for Tobacco to Maximise the Tax Revenue, Social Savings and the Net Monetary Benefits in Sri Lanka

**DOI:** 10.15171/ijhpm.2019.114

**Published:** 2019-12-01

**Authors:** Sathira Kasun Perera, Bharat Phani Vaikuntam, Denny John, Buddhika Senanayake

**Affiliations:** ^1^University of New South Wales, Sydney, NSW, Australia.; ^2^University of Sydney, Sydney, NSW, Australia.; ^3^Campbell Collaboration, New Delhi, India.; ^4^National Institute of Medical Statistics, New Delhi, India.; ^5^The University of Queensland, Brisbane, QLD, Australia.

**Keywords:** Optimum Tobacco Taxation, Tobacco Tax Sri Lanka, Tobacco Control

## Abstract

**Background:** Fiscal policy targeting tobacco control is identified as the most effective strategy for rapid control of tobacco use. An optimum fiscal policy to estimate the percentage taxation that will maximise the government tax revenue, social savings and the net monetary benefit has not been empirically designed before in Sri Lanka.

**Methods:** A model was developed using Microsoft Excel 2016, utilizing up-to-date published evidence on the cigarette sales, current fiscal policy, social cost of tobacco use, consumer response and the price elasticity of cigarettes. Univariate estimates on the expected revenue from tobacco tax, average annual social savings and the net monetary benefit were predicted for different levels of tobacco taxation. A deterministic sensitivity analysis was performed covering all possibilities. The percentage taxation maximizing the government tax revenue and the net monetary benefit were identified.

**Results:** It was estimated that a further 30% tax increase from the 2019 baseline will generate approximately LKR 3544 million per year of additional tax revenue for the government while saving LKR 28 069 million per annum as social savings. A fiscal elevation of 50% will produce identical annual tax revenue to that of 2018, while securing a social saving of more than LKR 47 600 million per annum. The maximum net monetary benefit is achievable at an overnight tax increase of 90% from the baseline, however with a short-term compromise in tax revenue.

**Conclusion:** The well-defined thresholds take tobacco taxation advocacy in Sri Lanka a step forward and will assist the government in taking an informed decision on its fiscal policy for cigarettes.

## Background

The use of tobacco products has been estimated to be the leading cause of burden of disease in developed countries. It is also the single most preventable cause of morbidity and mortality around the world, accounting for nearly 12% of all deaths among adults over 30 years of age.^1^ The health, social, and economic impact of smoking is substantial. The effect of productivity loss due to tobacco use is also significant as the tobacco mortality peaks in later adulthood owing to its associations with many chronic non-communicable diseases.^2^

The situation in lower-middle-income countries including Sri Lanka is no different. The economic burden of tobacco use was estimated to be above LKR 89 billion per year from a health systems perspective, including the household direct and indirect costs.^3^ If Sri Lanka can target to reduce tobacco consumption, it is possible to recover a significant amount of financial resources spent on managing complications of tobacco use.

The demand of tobacco, like any other usual ‘economic good’ responds to price changes relative to the price of other products, real income changes, and changes in tastes and preferences.^4,5^ Fiscal policies targeting tobacco control has by far been the most effective strategy for rapid cut-off of smoking rates. Tobacco specific excise taxes are more effective in achieving health gains compared to ad valorem taxes which are difficult to implement in complex tax systems.^6^ Evidence from both high and middle/lower income countries suggest that large increases in tobacco specific excise taxes to have a substantial and rapid effect on consumption.^7^ In many high-income countries, specific excise taxes account for around 50%-60% of the retail price, compared to 35% to 40% in middle/lower income countries.^8,9^ So, doubling the excise taxes can double the retail price in higher income countries, it would require even-higher tax increases to achieve a similar effect. Tax by weight is recommended as companies try to work around the tax if by per cigarette by increasing size of cigarette to king or super king.^10^ Careful regulation of imports and imposing excise duties help to control illegal imports of tobacco. The relative prices of alternative forms of tobacco and their relative health impacts significantly impact the overall tobacco consumption and health burden. Equivalent taxation of alternative products is required for the taxation to be effective in reducing health burden.^6^

Even though other control strategies like price promotion restrictions, minimum price laws and health promotion initiatives have proven effectiveness,^11^ fiscal interventions are easy to implement with an overnight impact on tobacco use, improving population health and reducing smoking related health risks. The World Health Organization Framework Convention on Tobacco Control identifies price and taxation-based interventions as a major tobacco control strategy.^12,13^ Fiscal interventions in many different countries across the globe have been observed to be highly effective in reducing consumption^14,15^ including in settings with similar socio-economic backgrounds to Sri Lanka.^16^ Simulations elsewhere have shown that even in a conservative scenario, increasing the price by 100% using fiscal measures maximizes revenues and reduce cigarette consumption.^17^

Currently, Sri Lankan fiscal policy on tobacco follows a lump sum tax imposed on the number of cigarettes sold. It is noted that there have been recent tax increases with evidence of reduction of tobacco consumption in the country.^18^ A tax is levied and paid on every cigarette manufactured in Sri Lanka. The rate is defined by the minister of finance, published in the Gazette. The tobacco tax is payable by the industry and the tax is computed based on the number of cigarettes manufactured at, and removed from, the factory at which such manufacture is carried on. Thus, the total amount of the tax payable is calculated on the total quantity of cigarettes removed from a factory.

The government tax revenue from tobacco sales in year 2017 was about LKR 88 billion.^18^ It is understood that even though there is a heavy social cost of tobacco use, being a third world country, the government depends on tax revenue to manage annual government expenditure. Thus, it is required to evaluate in economic terms, whether the current percentage taxation on tobacco products is the optimum rate that could maximize government excise tax revenue, while minimising the tobacco consumption and the social cost of tobacco use.

The objective of this analysis was to provide an empirically designed estimate of the optimum percentage of taxation that may be applied on tobacco products and to estimate the potential economic benefits of such an effective fiscal policy targeting tobacco control in Sri Lanka.

## Methods

A model was developed using Microsoft Excel 2016, utilizing up-to-date published evidence on the current status of cigarette sales in Sri Lanka, current fiscal policy on tobacco, social cost of tobacco use, effectiveness of tobacco control strategies, product substitution data, consumer response to cigarette price increase, and the price elasticity data of cigarettes (see Supplementary file 1).

### Data Inputs

Cigarette price for year 2019 was obtained from surveying 20 retail shops in Sri Lanka, where a uniform price was observed in all outlets. The information with regard to tobacco sales was obtained from the annual report of the Ceylon Tobacco Company^19^ which holds the monopoly of tobacco production and sales in Sri Lanka. Information on the current fiscal policy, fiscal limits on various tobacco products and the tax revenue was obtained from the Fiscal Management Report of the Ministry of Finance, 2017, Sri Lanka.^18^ Price elasticity of demand was obtained from studies conducted around the world.^20,21^ Social cost of tobacco use has been extensively quantified in a recent study conducted in Sri Lanka, based on the dose response data and tobacco attributable fraction of different tobacco related diseases. The direct and indirect costs of managing health conditions due to tobacco use including the health system costs, out of pocket expenditure and productivity loss has been quantified comprehensively in this recent estimation of tobacco related social costs conducted in Sri Lanka. Thus the annual social cost due to tobacco consumption estimated in this study was used as an input to the model.^3^ The data inputs and the range of values used in the model are summarized in Table 1.

**Table 1:** Data Inputs and Values Used in the Model Building Process

**Data Input**	**Range of Values Used**	**Reference**
Annual tobacco sales volume of individual tobacco products	Brand 1: 3154 million sticks	^19^
	Brand 2: 304 million sticks	
	Brand 3: 228 million sticks	
	Brand 4: 114 million sticks	
Market share of individual tobacco products	Brand 1: 83%	^19^
	Brand 2: 8%	
	Brand 3: 6%	
	Brand 4: 3%	
Price elasticity of demand of cigarettes	LMIC: - 0.53	^22^
	Population (LMIC): -0.74	^20^
	India: -0.65	^16^
	Youth (conditional): -2.11	^20^
	Adult: - 1.17	^21^
Price of different brands of cigarettes currently sold	Brand 1: LKR 65	Primary market data (November 2019)
	Brand 2: LKR 20	
	Brand 3: LKR 55	
	Brand 4: LKR 70	
Government revenue from tobacco taxation	LKR 87 400 million	^18,19^
Social cost of tobacco use in Sri Lanka (converted to 2019 value)	LKR 110 333 million	^3^
Industry claimed substitution factor for cheap alternatives (Beedi) – per 10% in taxes	1%-2%	^19^
Discount factor	0.03-0.06	^23^

Abbreviation: LMIC, low- and middle-income country.

### Model Building and Outcomes

Univariate estimates on the expected revenue from tobacco tax, average annual social cost savings and the net monetary benefits were derived for different predicted levels of tobacco taxation. Minimum and maximum values for all estimates were calculated in a deterministic sensitivity analysis covering all possibilities, to represent the uncertainty around the estimates.

Using economic evidence on fiscal policy and price elasticity, the suggested fiscal policy was developed, targeting tobacco control while optimizing government revenue. A graded analysis on the economic impact of the suggested fiscal policy was performed to inform all possible policy options for the government of Sri Lanka.

The graded increase of percentage taxation was applied on the current price of the commonly used tobacco products which govern more than 90% of the market share. A new range of price was estimated for each individual product.

Based on the price elasticity of demand for cigarettes, new demand curves for each product were derived. The changes to the quantity of sales were estimated for each product and the new sales volumes calculated. Predicted government tax revenue at each different level of taxation was calculated using the unit tax and the predicted quantity of sales at each level of taxation. The percentage taxation that will maximize the government tax revenue was identified. This was defined as the “tax revenue maximising cut off” for optimum tobacco taxation in Sri Lanka.

Social cost savings due to reducing tobacco consumption at each level of taxation was predicted using recently published Sri Lankan data on the social cost of tobacco use based on the dose response data and tobacco attributable fraction of different tobacco related diseases.

The level of taxation at which the government tax revenue remains unchanged from the baseline 2017 value, while producing greater social savings was identified. This was defined as the “revenue neutral cut off” from a government perspective.

The cost saving due to averted social cost was added to the annual increment of government tax revenue to calculate the net monetary benefit of implementation of the suggested fiscal policy at different levels of taxation from a government perspective. The percentage taxation that will maximize the net monetary benefit was identified. This was defined as the “net monetary benefit maximising cut off” for optimum tobacco taxation in Sri Lanka.

All calculations were based on the assumption that taxes are increased overnight from the baseline value up to the suggested new threshold. Tobacco industry’s own claim about substitution to other alternative unregulated products was also in cooperated to improve acceptability of the results from an industry perspective.

A deterministic sensitivity analysis was performed to account for the uncertainty around the price elasticity of demand of cigarettes and the substitution factor.

## Results

Table 2 depicts the model predicted tobacco sales volume, tax revenue, social savings and the net monetary benefit of increasing tobacco taxes at different percentages from the baseline tax.

**Table 2:** The Effect of Increasing Tobacco Taxation Expressed in Terms of Predicted Government Tax Revenue, Social Cost Savings and Net Monetary Benefit

**Level of Taxation (Actual Price of the 4 Brands of Cigarettes, in Order of Popularity LKR)**	**Total Predicted Annual Sales (Million Sticks)**	**Predicted Annual Tax Revenue (LKR Million)**	**Predicted Annual Social Savings (LKR Million)**	**Predicted Annual Net Monetary Benefit (LKR Million)**
Baseline (65, 20, 55, 70)	3800	115 810	0	0
10% increase (71.50, 22, 60.50, 77)	3492	116 990	8474	9654
20% increase (78, 24, 66, 84)	3253	118 899	18 271	21 361
30% increase (84.50, 26, 71.50, 91)	3015	119 354	28 069	31 613
40% increase (91, 28, 77, 98)	2776	118 354	37 866	40 410
50% increase (97.50, 30, 82.50, 105)	2537	115 899	47 664	47 754
60% increase (104, 32, 88, 112)	2298	111 991	57 461	53 642
70% increase (110.50, 34, 93.50, 119)	2059	106 628	67 259	58 077
80% increase (118, 36, 99, 126)	1821	99 810	77 057	61 057
90% increase (124.50, 38, 104.50, 133)	1582	91 538	86 854	62 582
100% increase (130, 40, 110, 140)	1343	81 811	96 652	62 653

**Figure 1:**
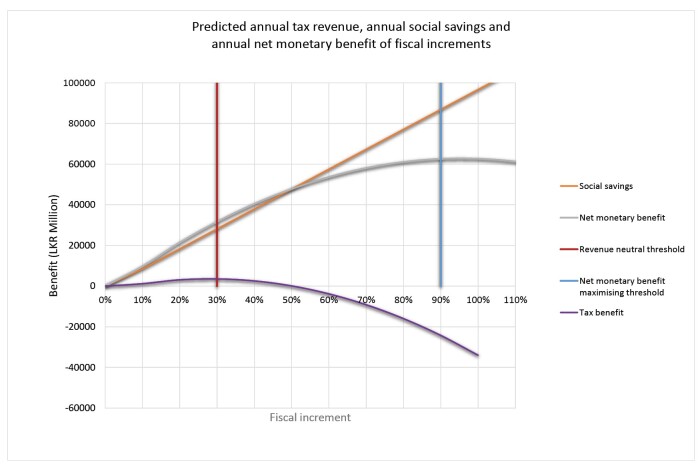
Predicting the Optimum Percentage of Taxation on a Cigarette Using the Tax Revenue, Social Cost Savings and net Monetary Benefit Predictions – Assuming Immediate Social Benefits.

**Figure 2:**
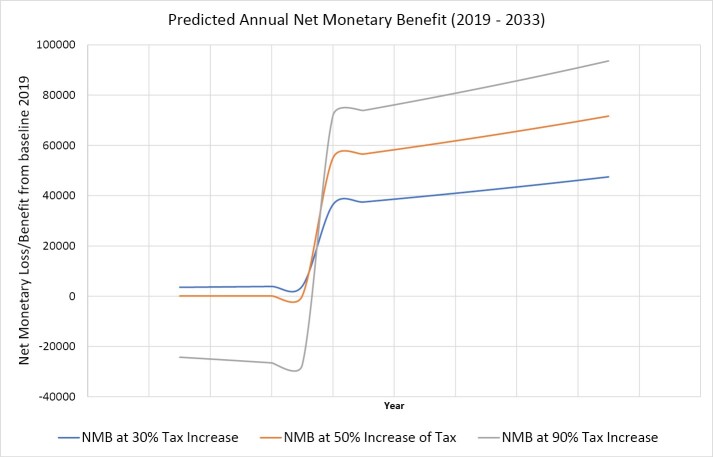
Predicting the NMB of Tobacco Tax Increase at 30%, 50%, and 80% From the Baseline – Assuming Delayed Social Benefits for 5 Years. Abbreviation: NMB, net monetary benefit.

The expected change in tax revenue, social cost and the predicted net monetary benefit over the range of fiscal inputs are depicted in Figure 1. The predictions for net monetary benefit at 30%, 50% and 80% tax increases from baseline are depicted in Figure 2, assuming a delayed social benefit for 5 years. It is evident that a 30% increase from baseline tax will maximize the government tobacco tax revenue while a 90% increase will maximize the net monetary benefits, assuming linear social savings from the origin. Thus, 30% was defined as the “tax revenue maximising cut-off” while 90% was considered the “net monetary benefit maximising cut-off.”

As depicted by the tax benefit curve in Figure 1, at about 50% increase from the baseline, the government tax revenue again crosses the baseline, indicating that the government can stay revenue neutral at this level, while eliciting a significant social saving of more than LKR 40 000 million per annum. Thus, 50% fiscal elevation was considered the “revenue neutral cut-off” of tobacco taxation.

The predicted net monetary benefit of the suggested fiscal changes is depicted at 30%, 50%, and 90% fiscal elevations over a period of 10 years, assuming a 5-year latent period for appearance of social cost savings. It is evident that a 30% increase in tobacco taxation will optimise government tax revenue while a 90% increase will have a negative effect on tax revenue in the short run. However, following the latent period of 5 years, the 90% increase in taxes produces the highest net monetary benefit when the social cost savings start to accumulate. Thus, in the long run, the tax increases less than 90% will be suboptimal.

Table 3 depicts the results of the deterministic sensitivity analysis with the range of expected values for the increase in tax revenue, social cost savings and the net monetary benefit at suggested 30% and 90% cut off values for tobacco tax increase. The model outputs were sensitive to the price elasticity of demand of cigarettes. Thus, a wide range of relevant inputs were used to increase the acceptability of results at different levels of price elasticity.

**Table 3:** Results of the Deterministic Sensitivity Analysis With the Predicted Annual Increase in Tax Revenue, Social Cost Savings and the Net Monetary Benefit at Suggested 30% And 90% Cut Off Values for Tobacco Tax Increase in Response to the Variability of Price Elasticity of Demand and Substitution Factor

**Input**	**Predicted Gain in Tax Revenue (LKR Mn)**		**Predicted Social Savings** **(LKR Mn)**		**Predicted Net Monetary Benefit** **(LKR Mn)**	
	**30% Tax Increase**	**90% Tax Increase**	**30% Tax Increase**	**90% Tax Increase**	**30% Tax Increase**	**90% Tax Increase**
Base case analysis Price elasticity -0.74, substitution factor 1%	3544	-24 272	28 069	86 854	31 613	62 582
Price elasticity of demand of cigarettes of -0.53	12 850	16 532	19 728	61 831	32 578	78 362
Price elasticity of demand of cigarettes of -0.65	7532	-6785	24 494	76 130	32 026	69 345
Substitution factor of 2%	7975	-4842	26 745	85 530	34 720	80 688

## Discussion

It is estimated that a 30% tax increase (to tax revenue maximising cut-off) from the baseline level of tobacco taxation will generate approximately LKR 3544 million per year of additional tax revenue for the government of Sri Lanka while saving LKR 28 069 million per annum as social cost due to reducing tobacco consumption. Thus, the net monetary benefit of increasing tobacco tax by 30% will exceed LKR 31 613 million per year. Considering the evidence that price elasticity of demand for cigarettes is greater among the youth and among the low-income groups, the actual benefit in terms of social cost saving could be greater than predicted.^24^ Raising tobacco taxes can have income distributional effects with disproportionate burden on lower income individuals in the short run, but these tobacco control measures are progressive and lead to reduction of social inequality in the long run.^25^ Past experience in different parts of the world shows that even with lower price elasticities such as -0.24, there could be greater fiscal gains with a considerable public health impact.^26^ Greater benefits have been demonstrated elsewhere.^27-29^ It has also been shown that greater tax hikes change the tobacco industry pricing strategy to increasing industry net-of-tax prices, as opposed to the strategy of shielding consumers in situations where there are smaller tax hikes.^26^

Gilmore et al^10^ found the pricing strategies of tobacco companies to be influential on the effectiveness of tobacco tax as the companies can potentially absorbing any tax increases for mid/ultra-low-price brands by over-shifting (increasing prices on top of tax) to premium brands in the United Kingdom. This could lead to bigger price gaps between premium and low-price brands, thereby enabling smokers to substitute to low-price brands. A similar effect has also been found by Nargis et al in Canada where a widened price differential between premium and discounted brands contributed to a higher share of discount brand cigarettes.^30^ Governments must monitor cigarette prices by price segment and consider industry pricing strategies in setting tobacco tax policies.

France and South Africa have halved the consumption in less than 15 years compared to nearly three decades time taken by the United States and the United Kingdom to halve the cigarette consumption.^31^ Between 1995–2005, France and South Africa introduced large tax increases annually resulting in tripling of inflation-adjusted cigarette prices, whilst doubling their inflation adjusted tobacco revenue despite halving the cigarette consumption.^32-34^ However, the lack of sufficient price rises between 2005 and 2010 have led to an increase in prevalence of smoking in France demonstrates the significance of sustained efforts to achieve robust tobacco control.

It could be noticed that at the proposed tax revenue maximising level of taxation in Sri Lanka, the tobacco sales will drop by an estimated 26%, shrinking the industry volume. The analysis of market trends on the response of the tobacco industry shows that in almost all scenarios, the industry diverts the tax increase to consumers, rather than absorbing it. The evidence also shows that the industry prefers to increase the price of cigarettes by a greater margin, to nullify the effect of reduced sales on the profit margin. Thus, it could be concluded that the proposed tax increase may not raise concerns for the viability of the industry, as often claimed by them.

It has also been shown that consumers are unlikely to substitute standard products of their choice, with substandard products like beedi.^35^ Even though, an assumption of non-substitution seems reasonable, a substitution factor of 1% per each 10% price increase on regulated tobacco products was considered for base-case analysis to allow for any possible margin of error. According to the tobacco industry’s own claim, the rise in beedi consumption has been 24% over a period of 10 years,^19^ while the price of regulated tobacco increased by more than 250%. Thus the assumption of 1% substitution to unregulated products per each 10% price increase on regulated tobacco, entertains the claim of the tobacco industry that the consumption of unregulated tobacco increases in response to tax hikes on regulated products.^19^ The price of the second most sold brand has a price of 20 LKR, while the top brand is 65 LKR. Since the great majority of the market is occupied by the premium brand, and the cheaper alternatives hold only a relatively small segment of the market, a significant trading down to cheaper brands is unlikely. However, a substitution factor of 2% for beedi was also considered in the sensitivity analysis to depict an extreme possibility, which also confirmed similar tax and social benefits. This effectively nullifies the claim of the industry that cigarette price increases lead to substantial loss of tax revenue to the government, while also depicting that the social savings also remain robust to the substitution factor.

It was also depicted that, at approximately 50% (revenue neutral cut-off) of fiscal elevation from the baseline 2017 tax thresholds, the government can stay revenue neutral (raise same tax revenue as of year 2017), while achieving about 50% of the maximum possible social savings. Thus, one may argue that this is the most practical and effective cut-off a government can choose as it produces significant social benefits without any impact on the tax revenue.

It is noteworthy that the maximum net monetary benefit is achievable at an overnight tax increase of 90% from the baseline (net monetary benefit maximising cut-off), assuming a price elasticity of -0.74. At the lower margin of the range of price elasticity used (-0.52), the model predicted that there will still be a gain in tax revenue of LKR 18475 million.

It could be concluded that the government of Sri Lanka is currently operating at a sub-optimal level of tobacco tax percentage, generating loss of excise tax revenue and greater opportunity costs in socio-economic, health and quality of life perspectives. Thus, it is recommended that the government revises its fiscal policy on tobacco based on any of the above identified cut-offs.

It is entirely a policy decision, which cut-off the government wants to choose. To summarize, the “tax revenue maximising cut-off” of 30% increase of tobacco taxation will maximise and the government tax revenue, still offering significant social savings. The “revenue neutral cut-off” of 50% increase will allow the government to enjoy the same tax revenue as of 2017, while achieving greater social savings. The “net monetary benefit maximising cut-off” of 90% increase will produce the optimum net monetary benefit for the country. The model predicted further that at the lower limits of price elasticity (-0.52), the government could expect to boost its excise tax revenue even at a percentage tax increase of 130%.

At tax elevations grater than 90%-130% however, it may be rational for the government to be prepared to mitigate the effects of short-term losses of tax revenue, while enjoying the greater social cost savings. This, if implemented may possibly represent one of the greatest empirically estimated health economic benefits due to any single public health policy intervention in the Sri Lankan history.

## Conclusion

Sri Lanka could move closer to its tobacco control targets by implementing the suggested fiscal policy, based on the above defined benchmarks. This can optimize the government tax revenue, significantly reduce social cost of tobacco consumption, shrink public spending for healthcare ailments associated with tobacco use and uplift the health-related quality of life of Sri Lankans. The tax rates must be adjusted annually to absorb the inflation and growth in income levels to continue the benefits of reduction in tobacco consumption. Since the calculations were based on 2019 prices of cigarettes, the model outputs need to be adjusted using the latest prices whenever the implementation decision is taken.

Given the transparent methodology and the overall positive outcome for the government and the public with minimal impact to industry existence, the suggested new fiscal limits may be acceptable in its scientific merits to all segments of the society including the tobacco industry.

## Ethical issues

Ethical clearance was not required as the article was an economic analysis based on published literature.

## Competing interests

Authors declare that they have no competing interests.

## Authors’ contributions

All authors contributed significantly, to the conception, design, analysis and interpretation of data, and the writing of the paper.

## Authors’ affiliations

^1^University of New South Wales, Sydney, NSW, Australia. ^2^University of Sydney, Sydney, NSW, Australia. ^3^Campbell Collaboration, New Delhi, India. ^4^National Institute of Medical Statistics, New Delhi, India. ^5^The University of Queensland, Brisbane, QLD, Australia.

## Supplementary files

Supplementary file 1 contains the static Excel model used in the base case analysis.

## Key messages

**Implications for policy makers**

- It is estimated that the use of tobacco contributes to a significant amount of disease burden and social costs worldwide. Taxation has been shown one of the most effective tobacco control strategies helping to minimise the social cost due to tobacco use. An optimum fiscal policy estimating the percentage taxation that will maximise the government tax revenue, minimise the social costs and produce the maximum net monetary benefit has not been empirically designed before in Sri Lanka. This study predicts the tax revenue, social cost and the net monetary benefit at different levels of tobacco taxation in Sri Lanka, from a government perspective.
- The current advocacy on this is not based on such well-defined cut off values, making it difficult for the policy-makers to take an informed decision on the exact thresholds to use in developing the fiscal policy. Taking advocacy on tobacco control in Sri Lanka to a higher level, the three well-defined thresholds identified for maximising tax revenue, remaining revenue neutral while maximising the social cost savings and for maximizing the net monetary benefit will assist the policy-makers to take an informed decision on its fiscal policy for cigarettes.

**Implications for the public**

This study may assist the public in re-establishing their perception that there is a social cost of smoking at population level and the resultant economic impact is not recoverable by the temporary gains in tax revenue. The public will also gain a better level of understanding on the need to implement taxation policies as a method of controlling tobacco use and the long-term benefits of such fiscal intervention. This will further strengthen and support the public perception in favour of raising tobacco taxes to empirically estimated cut-off values. The myth that “smoking a cigarette contributes to the tax revenue and indirectly to the economic development,” could be effectively nullified by understanding the core concepts of this modelling exercise in terms of negative net monetary benefit of increasing tobacco consumption.
